# Powering the plasma membrane Ca^2+^-ROS self-amplifying loop

**DOI:** 10.1093/jxb/ery179

**Published:** 2018-06-19

**Authors:** Igor Pottosin, Isaac Zepeda-Jazo

**Affiliations:** 1 Biomedical Center, University of Colima, Colima, Mexico; 2 School of Land and Food, University of Tasmania, Hobart Tas., Australia; 3 Food Genomics Department, University of La Ciénega Michoacán de Ocampo State, Sahuayo Mich, Mexico

**Keywords:** Anion channels, anion current, Arabidopsis, ascorbate, Ca^2+^ signaling, cytosolic Ca^2+^, hydroxyl radical, NADPH oxidase, ROS

## Abstract

This article comments on:

Makavitskaya M, Svistunenko D, Navaselsky I, *et al*. 2018. Novel roles of ascorbate in plants: induction of cytosolic Ca^2+^ signals and efflux from cells via anion channels. Journal of Experimental Botany 69, 3477–3489.

**Reactive oxygen species (ROS) and intracellular Ca^2+^ signaling interact with and amplify each other: the major ROS-producing plasma membrane enzyme NADPH-oxidase (NOX) is activated by cytosolic Ca^2+^, and in turn ROS activate Ca^2+^ influx across the plasma membrane. For the latter, NOX-produced superoxide anions need to be converted to hydroxyl radicals.** **Makavitskaya *et al.* (2018)** **have now demonstrated that salt stress promotes ascorbate efflux, which, via reduction of the apoplastic copper and iron ions, assists in the generation of hydroxyl radicals, thus inducing a rise in intracellular Ca^2+^ in the roots.**

Aerobic metabolism inevitably generates ROS, which could be very destructive to biomolecules and structures. Consequently, the main components of the antioxidant system appeared at almost the same moment as the ROS-producing ones, approximately 3.8–3.6 billion years ago (Inupakutika *et al.*, 2016). Stress-induced metabolic changes result in increased ROS production (oxidative stress component), which may be balanced or not by an increase in antioxidant activity, leading either to adaptation or death, respectively (Demidchik *et al.*, 2010; Morales and Munné-Bosch, 2016; Choudhury *et al.*, 2017). Compared to animals, plants generally show 10–1000 times higher resistance to ROS (as H_2_O_2_) (Haliwell and Gutteridge, 2015). At this point, ROS may be viewed as a burden, but the early evolutionary appearance of the NADPH-oxidases (NOX), a class of enzymes whose principal function is ‘deliberate’ ROS production, forces one to look on ROS generation from a different angle. NOX enzymes have been found in all multicellular eukaryotes, including plants, animals and fungi (Inupakutika *et al.*, 2016). The function of plant homologs (RBOH: Respiratory Burst Oxidase Homologs) was originally attributed to pathogen defense in the hypersensitive response. However, later on, roles of RBOH enzymes in growth and morphogenesis were established and, finally, in ROS and Ca^2+^ signaling in response to different abiotic stresses (reviewed by Baxter *et al.*, 2014; Kaur *et al.*, 2014; Choudhury *et al.*, 2017; see also Turkan, 2017).

## Plant NOX function and regulation: the role of intracellular Ca^2+^

Plant NOX are situated in the plasma membrane and, similarly to animal homologs, are formed by six transmembrane domains. The catalytic part consists of NADPH and FAD-binding domains in the N-terminus and two b-type hemes located between transmembrane domains III and V (Box 1). Unlike most of their animal counterparts (except mammalian Nox5 and Duox), plant NOX possess two specific Ca^2+^-binding sites (EF-hand motifs) in their N-terminus (Suzuki *et al.*, 2011). This underlies a specific type of plant RBOH regulation. Activation of plant RBOH critically depends on phosphorylation and cytosolic Ca^2+^ binding to the residues located in the N-terminus. Activation of Ca^2+^ influx conductance by HO^•^ (less commonly by H_2_O_2_), as for the first time demonstrated by Foreman *et al.* (2003), and activation of RBOH by inflowing Ca^2+^ provides a feed-forward mechanism for ROS and Ca^2+^ crosstalk (Box 1) (for early experimental demonstrations see Takeda *et al.*, 2008, Demidchik *et al.*, 2009). Crosstalk between ROS and Ca^2+^ signaling as well as ROS activation of Ca^2+^-permeable channels has also been shown in animal cells (Zhang *et al.*, 2016), but it seems to occur less frequently than in plants. In particular, a self-amplifying circuit of certain RBOH species combined with a Ca^2+^-permeable channel (of non-identified molecular nature or encoded by a member of the glutamate receptor or cyclic nucleotide-gated channel families) is involved in plant growth and development, phytohormone signaling, hypersensitive responses to pathogens and responses to abiotic stress (reviewed by Demidchik and Shabala, 2018).

**Box 1. Regulation of NOX activity and operation of the ROS-Ca^2+^ self-amplifying loop in plants**

Plant NADPH-oxidase (RBOH) is an integral plasma membrane protein which mediates a single-electron transmembrane transfer from NADPH to molecular oxygen, generating the superoxide-anion (O_2_^–^). The electron-transport chain from NADPH to O_2_ includes FAD and b-type hemes, located in the RBOH C-terminus and between transmembrane domains, respectively. The latter is converted to peroxide (H_2_O_2_) spontaneously or catalysed by superoxide dismutase (SOD). In the presence of reduced transient valency metals, copper (Cu^+^) or iron (Fe^2+^), H_2_O_2_ is reduced to the hydroxyl radical (HO^•^) by means of the Fenton reaction. Apoplastic Fe and Cu form part of low molecular weight complexes with organic acids or of metalloproteins, cell wall-bound peroxidases (POX, iron) and diamine oxidase (DAO, copper). Ascorbate in the apoplast is mainly exported from the cytosol.

Makavitskaya *et al.* (2018) recorded an anionic current mediating ascorbate efflux from roots for the first time. Ascorbate may directly interact with O_2_, generating H_2_O_2_, but it is far more essential for the generation of HO^•^, maintaining Fe, and especially Cu, in their reduced forms. In turn, HO^•^ activates multiple conductances across the plasma membrane, which mediate K^+^ efflux and Ca^2+^ influx. Incoming Ca^2+^ activates the RBOH synergistically by direct interaction with Ca^2+^-binding sites (EF-hands) in the N-terminus or via Ca^2+^-dependent phosphorylation of different residues either by CDPK or the CBL-CIPK couple (Qu *et al.*, 2017). This generates a positive feedback loop between generation of ROS and Ca^2+^ signals. Conversely, S-nitrosylation of the cysteine residue in the C-terminus was shown to inhibit AtRBOHD activity; as this residue is conserved in the other 18 AtRBOH members, such a regulatory mechanism may be hypothesized to be universal for the family (Kaur *et al.*, 2018). In Arabidopsis, NO generation is rapidly stimulated by H_2_O_2_, so it is tempting to think that this mechanism may serve as a feedback loop, terminating the RBOH-generated ROS signal.

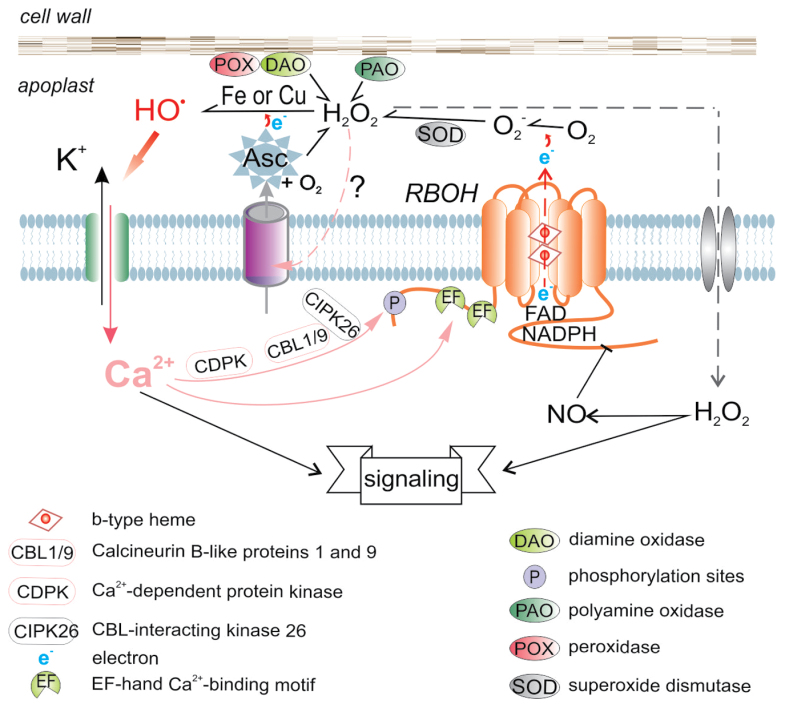

Different ROS species have different capabilities in the activation of Ca^2+^ conductance. For instance, extracellular H_2_O_2_ only induced activation of Ca^2+^-permeable channels in the root elongation zone, whereas HO^•^ also induced multiple conductances in the mature root zone (Demidchik *et al.*, 2007; Pottosin *et al.*, 2014). In the apoplast HO^•^ is generated through the Fenton reaction, via conversion of H_2_O_2_ by reduced transient valency metal ions, Fe^2+^ and Cu^+^. Copper is a much better catalyst of the Fenton reaction (reacting much more rapidly with H_2_O_2_) as compared to iron (Haliwell and Gutteridge, 2015). This is also confirmed by the data in the present study (Fig. 1B, Makavitskaya *et al.*, 2018). It should also be noted that standard redox potentials for the pairs Cu^2+^/Cu^+^ and Fe^3+^/Fe^2+^ differ by 600 mV, i.e. under physiological redox conditions copper will be several orders of magnitude more oxidized compared to iron. This difference will be compensated by reducing conditions in the presence of high concentrations of ascorbate (Asc).

## Export of reducing power: ascorbate efflux and signaling

Ascorbate is a very abundant cellular compound. About 90% of the available pool is cytosolic, yet it is exported to the apoplast across the plasma membrane and its concentration in this compartment may reach several millimolar (Akram *et al.*, 2017). Its role as a powerful antioxidant (H_2_O_2_ scavenger) in the cytosol is well established, but this process employs a sophisticated four-enzyme system, which may be less abundant in the apoplast (Podgorska *et al.*, 2017). As dicotyledonous plants can only transport iron across the plasma membrane in a reduced (Fe^2+^) form, export of ascorbate to the apoplast and its role in the reduction of Fe^3+^ to Fe^2+^ have been shown to be essential for iron uptake (Grillet *et al.*, 2014). Yet, ferrous compounds potentially possess pro-oxidant activity, as Fenton reaction catalysts assisting the generation of HO^•^. Therefore, rather than acting as an anti-oxidant, apoplastic ascorbate may be involved in HO^•^ production and the induction of HO^•^-sensitive Ca^2+^ conductance.

In line with this hypothesis, Makavitskaya *et al.* (2018) demonstrated that the addition of ascorbate to Arabidopsis roots induced cytosolic Ca^2+^ to increase in a dose-dependent manner. An ascorbate-induced increase in cytosolic Ca^2+^ was potentiated by externally applied Cu (as low as 0.001–0.01 mM) and Fe (1 mM) and suppressed by the HO^•^ scavenger thiourea, specific Cu and Fe chelators and low external Ca^2+^. Collectively, these results imply that an ascorbate-induced increase in cytosolic Ca^2+^ is caused by Ca^2+^ influx via HO^•^-activated plasma membrane conductance, where HO^•^ is generated by means of the Fenton reaction. Despite a higher affinity for copper as compared to iron, naturally occurring apoplastic Fe and Cu centers displayed comparable roles as Fenton catalysts in Arabidopsis roots. Their absence in cell wall-free preparations (root protoplasts) precluded a rise in ascorbate-induced cytosolic Ca^2+^. Up to this point, HO^•^ generation in the discussed work was achieved at least partially by the artificial, external application of Fenton reagents. It was previously demonstrated that acute salt stress provokes very significant generation of HO^•^ by Arabidopsis roots (Demidchik *et al.*, 2010). Application of the electron spin resonance (ESR) technique by Makavitskaya *et al.* (2018) allowed the sensitive detection of ascorbate (as the ascorbyl anion radical, Asc^•–^), released by Arabidopsis NaCl-stressed roots. Another important finding of this study was a direct recording of ascorbate efflux by means of the patch-clamp technique. Whereas import of the oxidized form of ascorbate for its recycling in the cytosol is relatively well understood (Akram *et al.*, 2017), to our knowledge this is the first time that the mechanism of ascorbate export to the apoplast has been addressed and successfully resolved. Ascorbate efflux measured as Asc^•–^ formation and whole-cell current (patch-clamp measurements) were suppressed by A9C with the same potency (Makavitskaya *et al.*, 2018), which suggests that the current is important for ascorbate efflux. The nature of this ionic current remains to be elucidated, though its rapid kinetics are reminiscent of the R-type anion current which plays an important role in stomatal closure. The major contributor to the R-type current in Arabidopsis guard cells is AtALMT12, which belongs to the ALMT (Aluminum-activated Malate Transporter) family. Another member of the ALMT family is predominantly expressed in Arabidopsis roots, with up-regulation by low external pH and, importantly, H_2_O_2_ (Sharma *et al.*, 2016). Thus, future experiments with AtALMT1 knockout mutants should enable its role in salt-induced ascorbate efflux from roots to be elucidated.

Another open question is how ascorbate export is stimulated under stress (i.e. how the activity of transporters is potentiated). One interesting possibility may be feed-forward regulation by ROS (Box 1). The ESR method, which detects oxidized Asc^•–^ as a measure of exported ascorbate, cannot be used for the evaluation of the ROS-induced ascorbate efflux because of the confounding effect of Asc^•–^ formed from direct ascorbate oxidation by added ROS. An alternative technique, however, based on measurements of ^14^C-labelled ascorbate efflux, has been used to demonstrate rapid (peak at 2 min) and massive (up to 20% of cellular ascorbate) ascorbate release from plant cell cultures, induced by 1–10 mM H_2_O_2_ (Parsons and Fry, 2010). The exact mechanism by which ROS stimulates ascorbate efflux is unknown. It may be a direct activation of the anionic transporter (which could be directly addressed in a patch-clamp study) or mediated by some ROS-associated factors (e.g. intracellular Ca^2+^ signaling) and/or be a consequence of electrocoupling with the ROS-induced K^+^ efflux (anion, and ascorbate in particular, efflux will be driven by K^+^ efflux for electroneutrality).

## Conclusion

The ROS-Ca^2+^ self-amplifying loop is gaining shape and has been shown to be involved in diverse plant responses. Ascorbate efflux could potentially intensify the operation of such a loop, assisting the formation of powerful hydroxyl radicals. However, whether such efflux is a common component of the self-amplifying mechanism and how it is co-ordinated in time and space with the operation of RBOH and/or ROS-activated plasma membrane channels will need to be addressed in future studies.
